# Immunogenicity of Influenza Vaccines: Evidence for Differential Effect of Secondary Vaccination on Humoral and Cellular Immunity

**DOI:** 10.3389/fimmu.2018.03103

**Published:** 2019-01-29

**Authors:** Sietske K. Rosendahl Huber, Marion Hendriks, Ronald H. J. Jacobi, Jan van de Kassteele, Jolanda C. Mandersloot-Oskam, Renée A. J. van Boxtel, Anne M. J. Wensing, Nynke Y. Rots, Willem Luytjes, Josine van Beek

**Affiliations:** ^1^Centre for Infectious Disease Control, National Institute for Public Health and the Environment, Bilthoven, Netherlands; ^2^Department of Medical Microbiology, University Medical Center Utrecht, Utrecht, Netherlands

**Keywords:** influenza vaccines, pandemic, T cells, humoral, cellular

## Abstract

While currently used influenza vaccines are designed to induce neutralizing antibodies, little is known on T cell responses induced by these vaccines. The 2009 pandemic provided us with the opportunity to evaluate the immune response to vaccination in a unique setting. We evaluated both antibody and T cell responses in a cohort of public health care workers (18–52 years) during two consecutive influenza seasons from 2009 to 2011 and compared the MF59-adjuvanted pandemic vaccine with the unadjuvanted seasonal subunit vaccine that included the pandemic strain [The study was registered in the Netherlands Trial Register (NTR2070)]. Antibody responses were determined in serum by a hemagglutination inhibition assay. Vaccine-specific T cell responses were evaluated by detecting IFN-γ producing peripheral blood mononuclear cells using whole influenza virus or vaccine-specific peptide pools as stimulating antigens. Mixed effects regression models were used to correct the data for influenza-specific pre-existing immunity due to previous infections or vaccinations and for age and sex. We show that one dose of the pandemic vaccine induced antibody responses sufficient for providing seroprotection and that the vaccine induced T cell responses. A second dose further increased antibody responses but not T cell responses. Nonetheless, both could be boosted by the seasonal vaccine in the subsequent season. Furthermore, we show that the seasonal vaccine alone is capable of inducing vaccine-specific T cell responses, despite the fact that the vaccine did not contain an adjuvant. In addition, residual antibody levels remained detectable for over 15 months, while T cell levels in the blood had contracted to baseline levels by that time. Hereby, we show that pandemic as well as seasonal vaccines induce both humoral and cellular responses, however, with a different profile of induction and waning, which has its implications for future vaccine design.

## Introduction

Worldwide, influenza virus causes seasonal epidemics resulting in a major social and economic burden and 290,000–650,000 deaths each year, while pandemic outbreaks affect the population to an even greater extent (1). Each individual acquires an influenza virus infection ~1–2 times every 10 years (2). During an infection, humoral and cellular immunity is acquired, which are able to clear the current infection, and protects the individual against subsequent infections. The homology between strains of influenza virus determines the level of protection: antibodies only provide neutralizing immunity against infection with homologous strains, while the cellular response is often directed to conserved regions of internal proteins of the virus and will therefore provide enhanced clearance of the virus regardless of homology of the surface proteins (3, 4).

When the population does not have protective antibodies to the surface proteins of influenza virus available, pandemics may occur (5, 6). In 2009, influenza A(H1N1)pdm09, a subtype from swine origin, was introduced into the human population. This was the first time in over 30 years that an influenza virus originating from an animal reservoir was able to transmit from human to human (7). Worldwide, pandemic vaccination campaigns were rapidly implemented to induce protective immunity and thereby prevent spread of the virus (8–10).

During this 2009 pandemic, it became clear that there was significant homology between the circulating pandemic H1N1 strain and the H1N1 strains that were circulating until 1957, as older individuals had pre-existing antibodies available (11–13). Individuals born after 1957 were expected to be naïve to this reintroduced H1N1 subtype and would consequently depend more on the activation of alternative arms of the immune system. Especially T cells provide a valuable contribution in limiting infection and disease during the emergence of new influenza virus strains by aiding in the development of specific antibodies or mediating cytotoxic effects on their own. For influenza virus infection, CD4^+^ and CD8^+^ T cells have been shown to limit disease, improve recovery of the infected individual and eventually clear the virus from the body (14–17).

In the pandemic setting, the oil-in-water emulsion MF59 was included as an adjuvant to reduce antigen dose, while the vaccine remained capable of inducing a seroprotective antibody titer (18). The exact mechanism through which MF59 acts is still poorly understood, but the induction of an early transient inflammation was shown to play an important role in recruitment of immune cells that take up and transport the antigen and MF59 to the local lymph nodes where the immune response is activated as reviewed by O'Hagan et al. (19) and Del Giudice et al. (20). MF59 was shown to activate CD4^+^ T cells, which play an important role in the induction of high affinity class switched antibodies (21–23). During the pandemic in the Netherlands, the MF59-aduvanted monovalent subunit vaccine (24) directed against the proteins hemagglutinin (HA) and neuraminidase (NA) of the pandemic strain, was offered to risk groups, pregnant women, and health care workers in a two-dose schedule (10, 25, 26).

In this study, we had the opportunity to analyze the immunogenicity of influenza vaccines and evaluate both the effect of an unusual two-dose influenza vaccine schedule and the effect of addition of the adjuvant MF59 to influenza vaccines on both the humoral and cellular immune response. In addition, this study encompassed the subsequent season in which the A(H1N1)pdm09 strain was included in the unadjuvanted seasonal subunit influenza vaccine together with a newly emerged H3N2 strain. This allowed us to compare the immunogenicity of an adjuvanted subunit vaccine vs. an unadjuvanted subunit vaccine and to analyze the potential booster effect of previous vaccination with the A(H1N1)pdm09 strain. Analysis of immunogenicity was performed by measuring antibody responses and by investigating vaccine-specific T cell responses. Hereby, this study contributes to expanding knowledge on the humoral and cellular immunity in response to adjuvanted and unadjuvanted influenza subunit vaccination.

## Materials and Methods

### Study Cohort

A multicenter, non-randomized, controlled, open-label trial was conducted during the influenza pandemic and the subsequent influenza season from October 2009 until May 2011. Humoral and cellular immune profiles after vaccination with MF59-adjuvanted and unadjuvanted subunit vaccines were measured, modeled, and evaluated.

**Figure 1:**
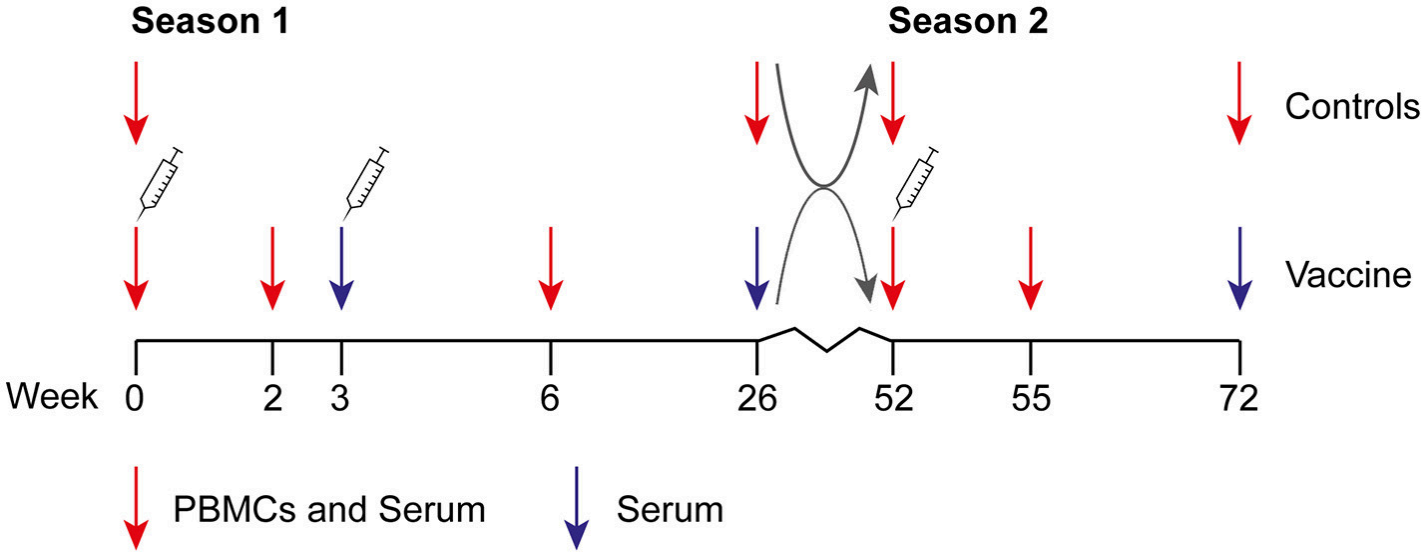
Design of the clinical study. The study was performed during two consecutive influenza seasons. During season 1 (2009–2010), individuals were vaccinated at the start of the study and 3 weeks later with the MF59-adjuvanted subunit vaccine. Three weeks before the start of the study or at week 6, an optional seasonal 2009–2010 vaccination was allowed. Gray arrows depict reallocation in control and vaccine groups. During season 2 (2010–2011), individuals in the vaccine group received the unadjuvanted seasonal 2010–2011 subunit vaccine at week 52. An unvaccinated control group was included in both seasons. Study participants could change between vaccine and control group at the start of season 2, resulting in a vaccine (V_1_) and control (C_1_) group in season 1 and vaccine-vaccine (V_1_V_2_), vaccine-control (V_1_C_2_), control-control (C_1_C_2_), and control-vaccine (C_1_V_2_) groups in season 2.

Healthy adults (18–52 years) were recruited among workers of public health institutions in the Netherlands. Exclusion criteria were: previous diagnosis with A(H1N1)pdm09 or fever within the last 2 weeks before the start of the study, any history with serious allergic reaction to vaccine components, and factors that might interfere with blood collection or immunological analysis. Study participants had the choice to be vaccinated or not in both seasons, independent of their choice in the previous season, resulting in a vaccine (V_1_), and control (C_1_) group in season 1 (2009–2010) and vaccine-vaccine (V_1_V_2_), vaccine-control (V_1_C_2_), control-control (C_1_C_2_), and control-vaccine (C_1_V_2_) groups in season 2 (2010–2011) (Figure 1). Participants were monitored for influenza virus infection: upon self-reported influenza-like illness (ILI) according to the Pel criteria (27), a nasopharyngeal swab was obtained within 72 h after onset of symptoms. Participants with a laboratory-confirmed influenza virus infection within 7 days of primary vaccination were excluded from the analysis.

The protocol was approved by the medical ethical reviewing committee (Central Committee on Research Involving Human Subjects) of the Netherlands and the study was conducted in accordance with Good Clinical Practice and the principles of the Declaration of Helsinki. Written informed consent was obtained from each participant. The study was registered in the Netherlands Trial Register (NTR2070).

### Vaccines

Participants of the vaccine group of season 1 received two doses of MF59-adjuvanted monovalent A(H1N1)pdm09 subunit vaccine (pH1N1 vaccine; Focetria, Novartis, Italy) with a 3-week interval (Figure 1). Seasonal influenza vaccination with trivalent subunit vaccine Influvac 2009–2010, containing A/Brisbane/59/2007 (H1N1), A/Brisbane/10/2007 (H3N2), and B/Brisbane/60/2008 (Solvay, The Netherlands) was optional, but if so, had taken place at least 3 weeks prior to the study or at week 6. In season 2, participants in the vaccine group were vaccinated with trivalent subunit vaccine Influvac 2010–2011 containing A/California/7/2009 (H1N1), A/Perth/16/2009, and B/Brisbane/60/2008 (Solvay, The Netherlands).

### Blood Collection

Blood was collected before vaccination, 2 and 3 weeks after the first dose, 3 weeks after the second dose and at the end of season 1 (Figure 1). During season 2, blood was drawn before and 3 weeks after vaccination and at the end of this influenza season. Blood was collected for PBMC isolation and serum at all time points, except for 3 weeks after the first dose and at the end of both seasons when only blood for serum was collected. Blood of individuals in the control group was collected for serum and PBMC isolation at the start and at the end of both seasons. Serum was stored at −20°C until analysis. PBMCs were isolated by Ficoll (Lymphoprep, Axis-Shield, Norway) density gradient centrifugation and stored at −135°C.

### Virus Strains

Influenza A/California/07/09 (A(H1N1)pdm09) virus was kindly provided by Institute Pasteur, France. A/Perth/16/2009 (H3N2) was obtained through NIBSC (United Kingdom). Viruses were grown on Madin-Darby Canine Kidney (MDCK) cells. Sequences of HA and NA proteins of these strains were obtained from GenBank (Protein accession numbers ACP44189, ACQ63272, ACS71642, and AHX37631).

### Hemagglutination Inhibition (HI) Assay

HI assays against wild type virus were performed in duplicate according to standard methods of the World Health Organization (WHO) at Viroclinics (Rotterdam, the Netherlands) and at the RIVM (28, 29). In short, a dilution series of cholera filtrate-treated serum samples was incubated with four Hemagglutinin Units (HAU) influenza virus for 20 min and 0.25% (v/v) turkey erythrocytes for 30 min and scored for agglutination. An HI titer of 40 or higher was defined as protective antibody level (29).

### Enzyme-Linked Immunospot (ELISpot) Assays

PVDF-membrane plates (Millipore Corporation, USA) were ethanol-activated, coated with 5 μg/mL 1-D1K anti-IFN-γ antibody (Mabtech Ab, Sweden), and incubated O/N at 4°C. Plates were blocked with AIM-V medium (Thermo Scientific, The Netherlands) containing 2% human AB serum (Sigma, MO, USA). For analysis of responses to the vaccine strains, 2^*^10^5^ PBMCs per well were incubated in AIM-V medium containing 2% human AB serum and stimulated with influenza virus in duplicate at a MOI of 4, mock (cell supernatant), or 1 μg/mL Staphylococcus Enterotoxin B (SEB) (Sigma, Germany). Analysis of the vaccine-specific antigens was performed in duplicate by stimulation of 4^*^10^5^ cells per well with 1 μg/mL of a peptide pool spanning the entire HA or NA protein of A(H1N1)pdm09 or A/Perth/16/2009 (JPT peptide Technologies, Germany). Per protein, 15-mer peptides with 11-mer overlap were pooled and dissolved in DMSO. In the negative control wells, DMSO was added to the medium. After an incubation period of 18 h, plates were washed with phosphate buffered saline (PBS) 0.2% triton-x100 to inactivate the virus, and detection IFN-γ biotin-labeled antibody 7-B6-1 (Mabtech Ab, Sweden) was added at 1 μg/mL in PBS 0.5% FCS (HyClone Thermo Scientific, USA) for 2 h at room temperature (RT). Plates were washed and incubated with streptavidin-alkaline phosphatase in PBS 0.5% FCS for 1 h at RT. After washing the plates, 100 μL NBT/BCIP solution (Sigma, MO, USA) was added. Color reaction was stopped by washing the plates with tap water. Plates were dried O/N at RT and spots were counted with A.EL.VIS reader (A.EL.VIS GmbH, Germany).

### Statistical Analysis

Mann Whitney U and Pearson Chi Square tests were applied to analyze the characteristics of the cohort, as indicated in the Results section. Statistical significance was defined as a *P* ≤ 0.05 and statistical analysis was performed with SPSS 19.0 statistical software program for Windows. Data from HI analyses were 2-log transformed and tested with a paired *T* test for longitudinal samples of the participants of the same group and with an unpaired *T*-test with Welch's correction for samples of participants of different groups. Data from ELISpots were corrected by deducting the appropriate negative controls and tested for significance with the Wilcoxon matched-pairs signed rank test, using GraphPad Prism 7.04 software.

To account for individual variation and other confounding factors, results from HI assays and ELISpot assays with virus-stimulated PBMCs were analyzed statistically using the mixed effects regression models to quantify differences in immune responses between vaccinated and unvaccinated groups (30, 31). A gaussian mixed effects regression model was used to analyze data from the HI assays and a mixed effects negative binomial regression model was used to analyze the data from ELISpot assays. The negative binomial distribution was used to describe the number of spots as counted per well in the ELISpot assay, while the underlying spot rates were modeled by the regression model. SEB counts were included in the regression model as denominator in the so-called offset term, i.e., if the spot rate is constant, higher SEB spot counts will automatically result in higher virus specific spot counts. For both models, possible confounders such as sex, vaccination history, and earlier influenza infections were taken into account as categorical variables and age was entered in the model as a natural cubic spline curve. A log-link function was used to relate the response rate with these fixed effects. To account for variation between participants, a random intercept was included in the model (32). Differences between groups are presented as either GMT ratios for HI data or relative rates for ELISpot data, including 95% confidence intervals, and *P*-values. The Holm adjustment is applied to correct for multiple testing. These analyses were performed in R using the R-INLA package (33, 34).

## Results

### Study Cohort

**Figure 2:**
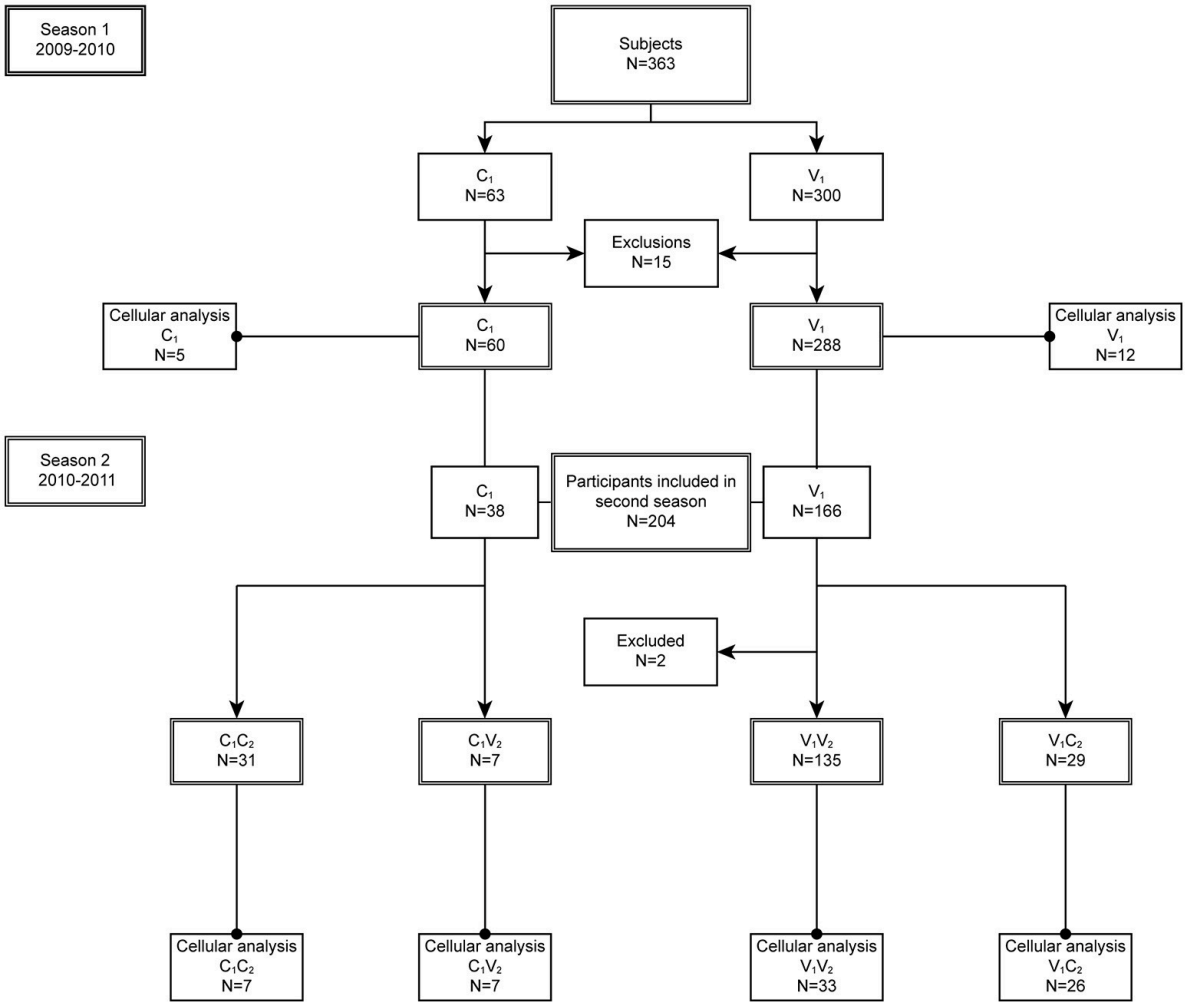
Study disposition. In season 1 (2009–2010), 15 participants were excluded from the per protocol analysis: eight were lost to follow up, two due to occupational vaccination while in the control group, four only received the first dose of the vaccination, and one individual was too old. In season 2 (2010–2011), two individuals were excluded from the per protocol analysis: one individual withdrew consent, one was excluded due to use of corticosteroids. All participants of the per protocol group were included in the humoral analysis, while a subgroup was included in the cellular analysis.

In season 1 during the pandemic of 2009, 348 individuals were included in the per protocol analysis of the study of whom 288 chose to be vaccinated (V_1_) and 60 chose not to be vaccinated (C_1_) (Figures 1, 2). In season 2, 202 individuals participated again and chose to be vaccinated or not, independent of their choice in season 1. This resulted in four different groups: 135 individuals remaining in the vaccine group (V_1_V_2_), 29 individuals switching from the vaccine to the control group (V_1_C_2_), 31 individuals remaining in the control group (C_1_C_2_), and 7 individuals switching from control to the vaccine group (C_1_V_2_). Baseline characteristics of the study participants are described for season 1 (Table 1A) and season 2 (Table 1B). Vaccination history of all participants was recorded, which shows that the number of frequent vaccinees was higher in the vaccination groups, which can be explained by work-related mandatory vaccination (Tables 1A,B).

**Table 1A:** Baseline characteristics season 1 (2009–2010).

		**C_**1**_ group (*n* = 60)**	**V_**1**_ group (*n* = 288)**	***P*-value**
Mean age (years) [range (years)]		39.1 *(25–52)*	39.0 *(19–52)*	n.s.
Gender (%)	Male	28.3	43.4	0.03
	Female	71.7	56.6
Any previous influenza vaccination (%)		20.0	56.6	0.001
Seasonal vaccination 2009–2010 before trial (%)		8.3	24.3	0.006
Seasonal vaccination 2009–2010 at week 6 (%)		5.0	34.7	n.s.
Laboratory-confirmed Influenza A infection (N)		0	0

*The italic values depicted are in years*.

**Table 1B:** Baseline characteristics season 2 (2010–2011).

		**C_**1**_C_**2**_ group (*n* = 31)**	**C_**1**_V_**2**_ group (*n* = 7)**	**V_**1**_V_**2**_ group (*n* = 135)**	**V_**1**_C_**2**_ group (*n* = 29)**	***P*-value**
Mean age (years) [range (years)]		39.7 *(27–52)*	39.6 *(25–52)*	41.0 *(19–52)*	39.1 *(23–52)*	n.s.
Gender (%)	Male	32.3	14.3	48.9	31.0	0.06
	Female	67.7	85.7	51.1	69.0
Seasonal vaccination 2009–2010 (%)		3.2	85.7	76.3	20.7	0.0001
Any previous influenza vaccination (%) 2009		6.5	100	80.7	34.7	0.0001
Laboratory-Confirmed Influenza A infection (N)		1		1	

*The italic values depicted are in years*.

### One Dose of the pH1N1 Vaccine Induced Adequate Antibody Responses

**Figure 3:**
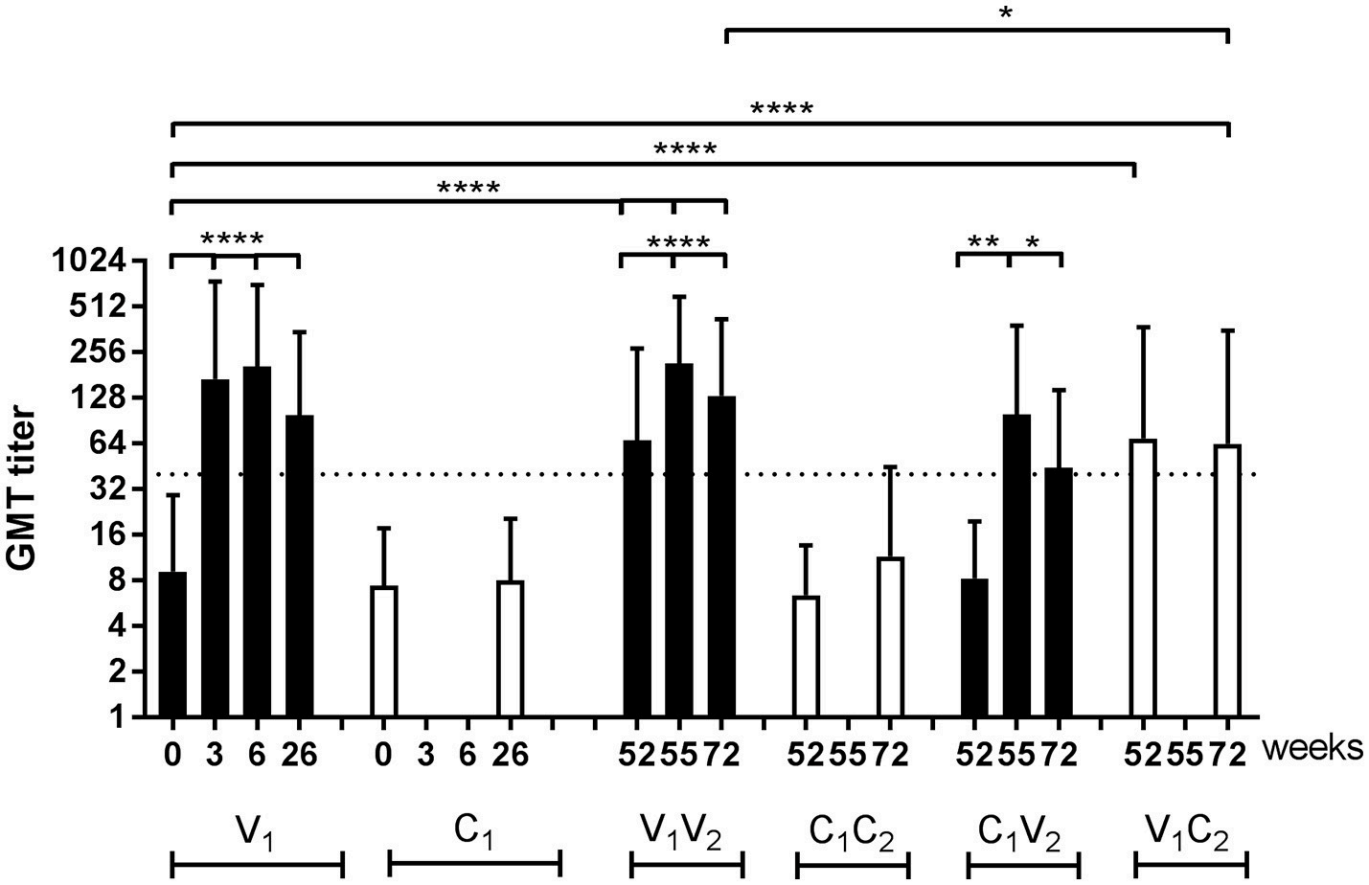
HI titers of influenza virus-specific antibody responses. Geometric mean titer (GMT) with SD of A(H1N1)pdm09-specific antibodies in vaccinated individuals and individuals of the control group of the per protocol group during season 1 and season 2. Antibody responses were tested with paired *T* test for longitudinal samples of individuals in the same group and unpaired *T* test with Welch's correction for analysis of samples from different groups. ■ vaccinated □ controls ^**…**^ protective antibody level of 40 ^*^P < 0.05, ^**^P < 0.01, ^****^P < 0.0001, N.A. not applicable.

Antibody responses to A(H1N1)pdm09, as analyzed by hemagglutinin inhibition (HI) analysis, are depicted for all groups during both seasons (Figure 3). First, the data were statistically analyzed longitudinally in a pairwise-manner within the different groups. One vaccination with the adjuvanted pH1N1 vaccine resulted in a significant induction of influenza virus-specific antibodies at week 3 (*P* < 0.0001; V_1_). This response could be boosted by a second dose of the adjuvanted vaccine 3 weeks later at week 6 (*P* < 0.0001; V_1_ week 6 vs. week 3) (Figure 3). By week 26, the antibody levels induced by the two doses of vaccine had waned (*P* < 0.0001), although antibody levels were still significantly higher than baseline (*P* < 0.0001). In the controls (C_1_), no significant antibody induction was observed (Figure 3).

### Residual Antibody Levels Were Boosted by Seasonal Vaccine

At the start of season 2, at week 52, the antibody levels in vaccinated individuals were still significantly higher compared to the baseline levels in season 1 (*P* < 0.0001; V_1_V_2_ and V_1_C_2_ vs. V_1_). Vaccination with an unadjuvanted seasonal subunit vaccine in season 2 resulted, both in the V_1_V_2_ and the C_1_V_2_ group, in a significant increase in antibody levels (*P* < 0.0001 and *P* = 0.01) (Figure 3). This was also the case for the antibody responses to the H3N2 vaccine strain that was introduced into the vaccine during season 2 (*P* < 0.0001; Supplemental Figure 1A). At the end of the study, at week 72, individuals in the V_1_V_2_ group who received both adjuvanted, and unadjuvanted vaccination in both seasons, ended up with a higher antibody level compared to V_1_C_2_ individuals who only received an adjuvanted vaccination in the pandemic season as statistically analyzed in an unpaired manner (*P* = 0.04) (Figure 3). Between the C_1_V_2_ and V_1_C_2_, no significant difference in antibody level was observed (Figure 3). These results show that the antibody response lasts at least 15 months and indicates an advantage of annual vaccination with the same vaccine strain on antibody levels.

### pH1N1 Vaccine-Induced Cellular Immune Responses

**Figure 4:**
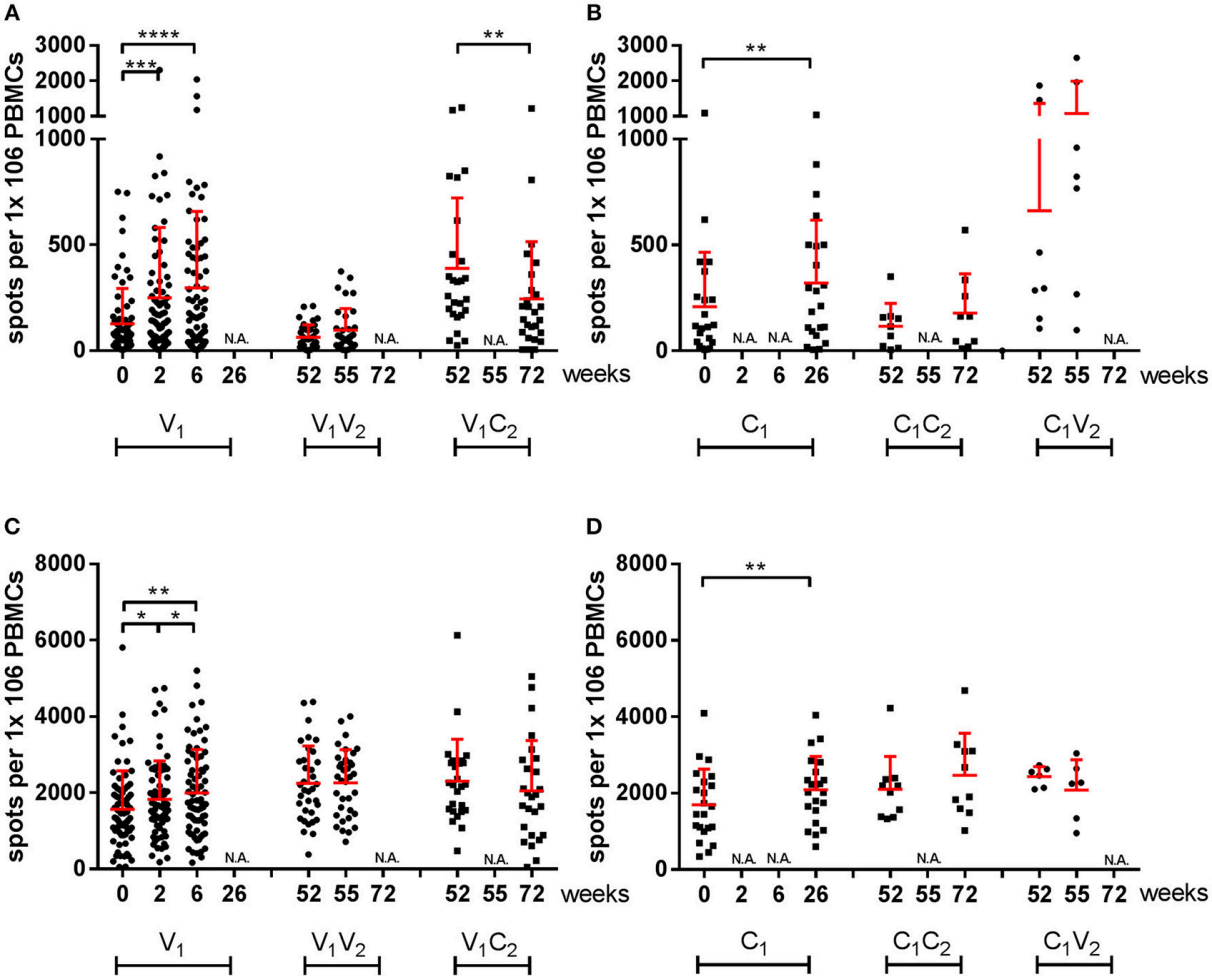
IFN-γ-specific responses of influenza virus-stimulated and SEB-stimulated PBMCs by ELISpot. Spots per million PBMCs of A(H1N1)pdm09 virus-specific **(A,B)** and SEB-induced **(C,D)** IFN-γ responses by ELISpot in vaccinated individuals and individuals of the control group during season 1 and season 2. In red the mean and SD of each data set is depicted. ELISpot data were analyzed with Wilcoxon matched-pairs rank test. •vaccinated, ■ controls ^*^P < 0.05, ^**^P < 0.01, ^***^P < 0.001, ^****^P < 0.000,1 N.A. not applicable.

Subsequently, we investigated the induction of T cells by a different number of doses of the pandemic and seasonal vaccines by stimulation of PBMCs with homologous virus in a subset of participants using IFN-γ ELISpots. These participants were selected to represent the four subgroups and the large range of observed antibody responses. First, the cellular immune responses to the virus strains after pH1N1 vaccination were analyzed in a pairwise-manner (Figures 2, 4A,B). A significant increase in IFN-γ spots was observed in the V_1_ group 2 weeks after the first dose of the pandemic vaccine (*P* = 0.003) (Figure 4A). A second dose of the vaccine did not result in a further increase in the influenza virus-specific response at week 6 (*P* = NS; V_1_). In season 2, a trend of induction of IFN-γ spots was observed in the C_1_V_2_ group (Figure 4B) and no induction was observed in the V_1_V_2_ groups after seasonal vaccination or in the V_1_C_2_ controls. Interestingly, we observed a significant induction of IFN-γ spots to the H3N2 strain in the V2 group after seasonal vaccination (Supplemental Figure 1B).

### Mixed Effects Regression Models for Controlled Analysis of Immune Responses

The data presented here was also corrected in the model for age, sex, and previous vaccination. We applied the mixed effects regression models to both datasets, a Gaussian mixed effects regression model for the serological responses and a mixed effects negative binomial regression model for the cellular responses. Thereby both datasets are depicted as relative inductions and reductions of the responses to enable comparisons between the two arms of the immune system. In the analysis of the cellular response, we included two sets of controls: an unvaccinated control group to evaluate asymptomatic exposure to the influenza virus, and SEB as an assay control for cell quality. In some of the controls (C_1_) (Figure 4B) and the SEB-stimulated samples (Figures 4C,D; Supplemental Figure 1C) we observed an increase in spots over time. We analyzed the data in a mixed effects negative binomial regression model that corrects for the observed overall increase in SEB responses for the cellular immune response of the participants over time.

**Figure 5:**
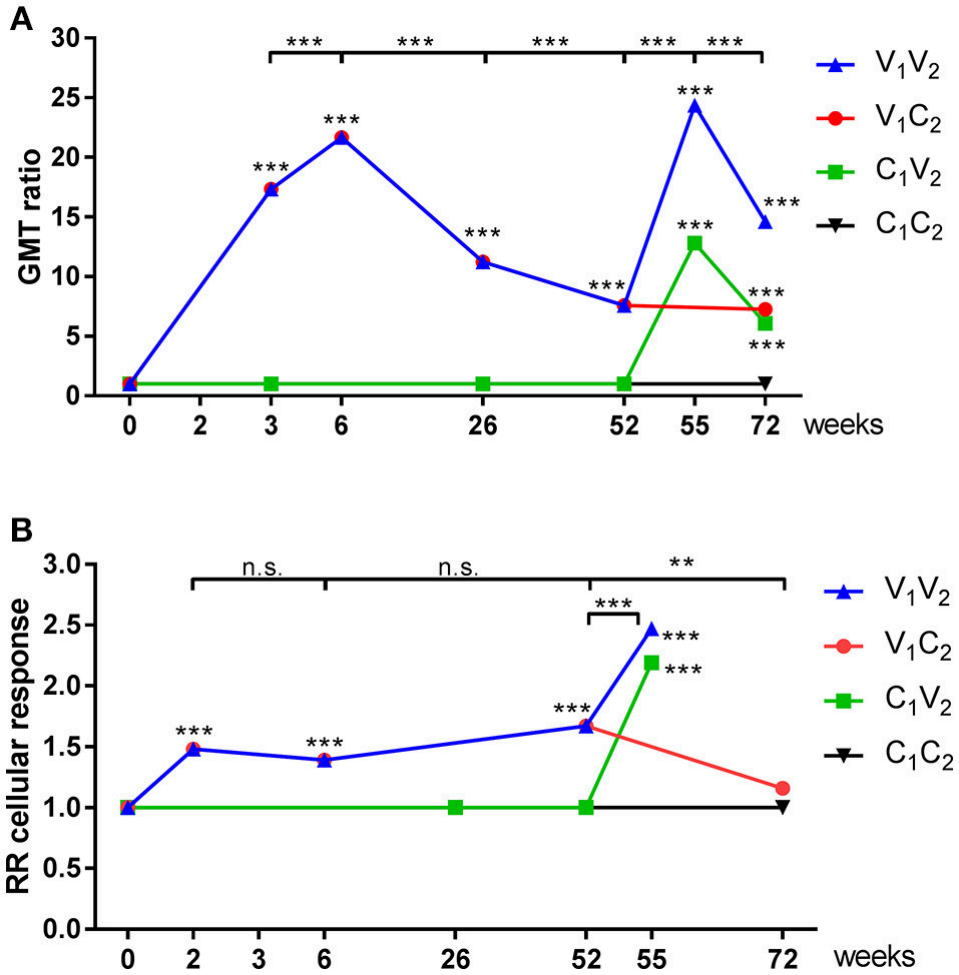
GMT ratios of serological and relative response rates of cellular immune responses. GMT ratios **(A)** en Relative response rates **(B)** were calculated by mixed effects regression models for antibody titer **(A)** and IFN-γ spots **(B)** for vaccinated individuals and individuals of the control group during season 1 and season 2. Statistical analysis of a time point compared to baseline are depicted in the graph, while analysis between time points is depicted above the graphs. ^**^P < 0.01, ^***^P < 0.001, ^****^P < 0.0001.

In line with Figure 3, we observed an induction of antibodies in season 1 during the pandemic (*P* < 0.001; Figure 5A; Supplemental Tables 1A–D) and also an induction of IFN-γ spots (*P* < 0.001 Figure 5B; Supplemental Tables 2A–C) 2 weeks after the first vaccination at week 0. The second pH1N1 vaccination in season 1 at week 3 resulted in a boost of the antibody response (*P* = 0.001) and waning at the end of that season (*P* < 0.001) (Figure 5A). No additional response was observed at the cellular level and the level remained the same for the rest of that season (*P* = NS; Figure 5B). In season 2, antibody and IFN-γ spots specific for A(H1N1)pdm09 were induced by the seasonal vaccine in both the V_1_V_2_ (*P* < 0.001; *P* = 0.001, respectively), and the C_1_V_2_ groups (*P* < 0.001; *P* < 0.001, respectively). Induction of H3N2-specific antibodies and cells was observed in individuals vaccinated with the seasonal vaccine (V_2_), confirming that not only the pandemic vaccine but also the seasonal vaccine is capable of inducing a vaccine-specific cellular response (GMT ratio 4.7 [3.9–5.6; *P* < 0.001]; Supplemetal Table 1E; RR 1.9 [1.6–2.2; *P* < 0.001; Supplemental Table 2D]). An additional effect of seasonal vaccination on the antibody levels was observed in the V_1_V_2_ group compared to the individuals of the C_1_V_2_ group who were only vaccinated in season 2 (*P* = 0.028; Figure 5A, Supplemental Table 1C), while no significant difference was observed in the IFN-γ spot level of the individuals in the V_1_V_2_ group compared to those in the C_1_V_2_ group (*P* = NS; Figure 5B, Supplemental Table 2C). These results indicate that previous adjuvanted pH1N1 vaccination does not provide an advantage on cellular immunity. Finally, the cellular response of individuals that switched to the control group in season 2 (V_1_C_2_) had decreased to the baseline level of season 1 by week 72 (*P* = NS; Figure 5B and Supplemental Table 2B).

### pH1N1 and Seasonal Vaccine Induce HA and NA-Specific Responses

**Figure 6:**
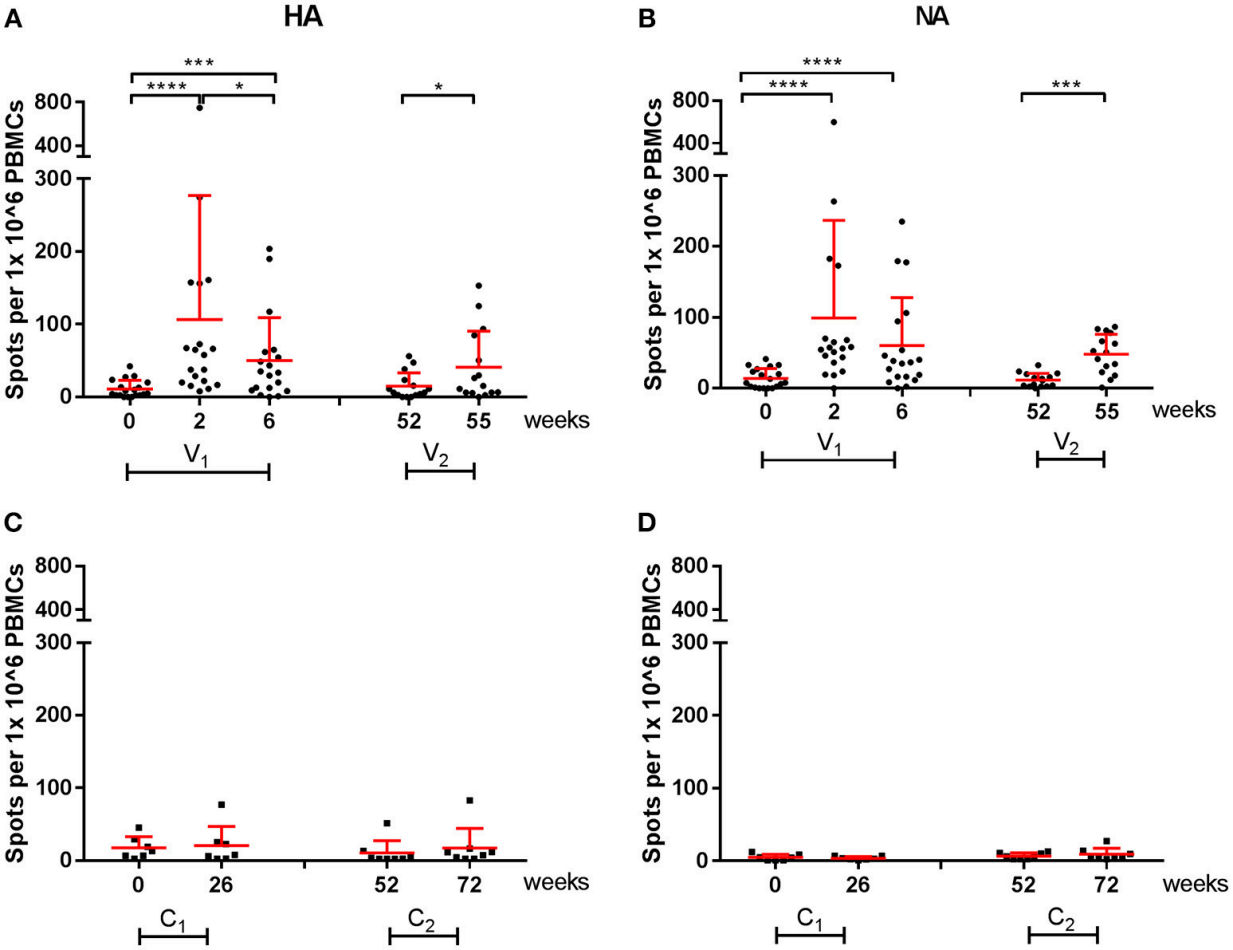
A(H1N1)pdm09 virus-specific cellular responses in season 1 and 2. Responses against HA **(A)** and NA **(B)** peptide pools were measured with an IFN-γ ELISpot in individuals of the vaccine group. In addition, responses against HA **(C)** and NA **(D)** were measured on controls. In red the mean and SD of each data set is depicted. ELISpot data were analyzed with Wilcoxon matched-pairs rank test ^*^P < 0.05, ^***^P < 0.001, ^****^P < 0.0001.

All cellular responses described above were analyzed by stimulation of PBMCs with live homologous virus. As the active substance of the vaccines are the HA and NA proteins of the influenza virus, we postulated that vaccine-induced responses described after virus stimulation were directed to the HA and NA proteins. To confirm this hypothesis, responses specific for the vaccine strains were further analyzed in an IFN-γ ELISpot by stimulation of PBMCs with HA or NA peptide pools. In Figure 6, responses to the HA- and NA-peptide pools of A(H1N1)pdm09 are depicted. After one dose, there was a significant increase in cellular responses to HA (*P* < 0.0001), which were not boosted by the second dose in season 1 (Figure 6A). Similar observations were made for NA protein (*P* < 0.0001; Figure 6B). Responses were also tested in a control group consisting of individuals who had not reported an infection, did not receive a vaccination and did not have antibody levels toward HA (≤ 5 HI titer). These individuals did not have a measurable change in responses (Figures 6C,D).

**Figure 7:**
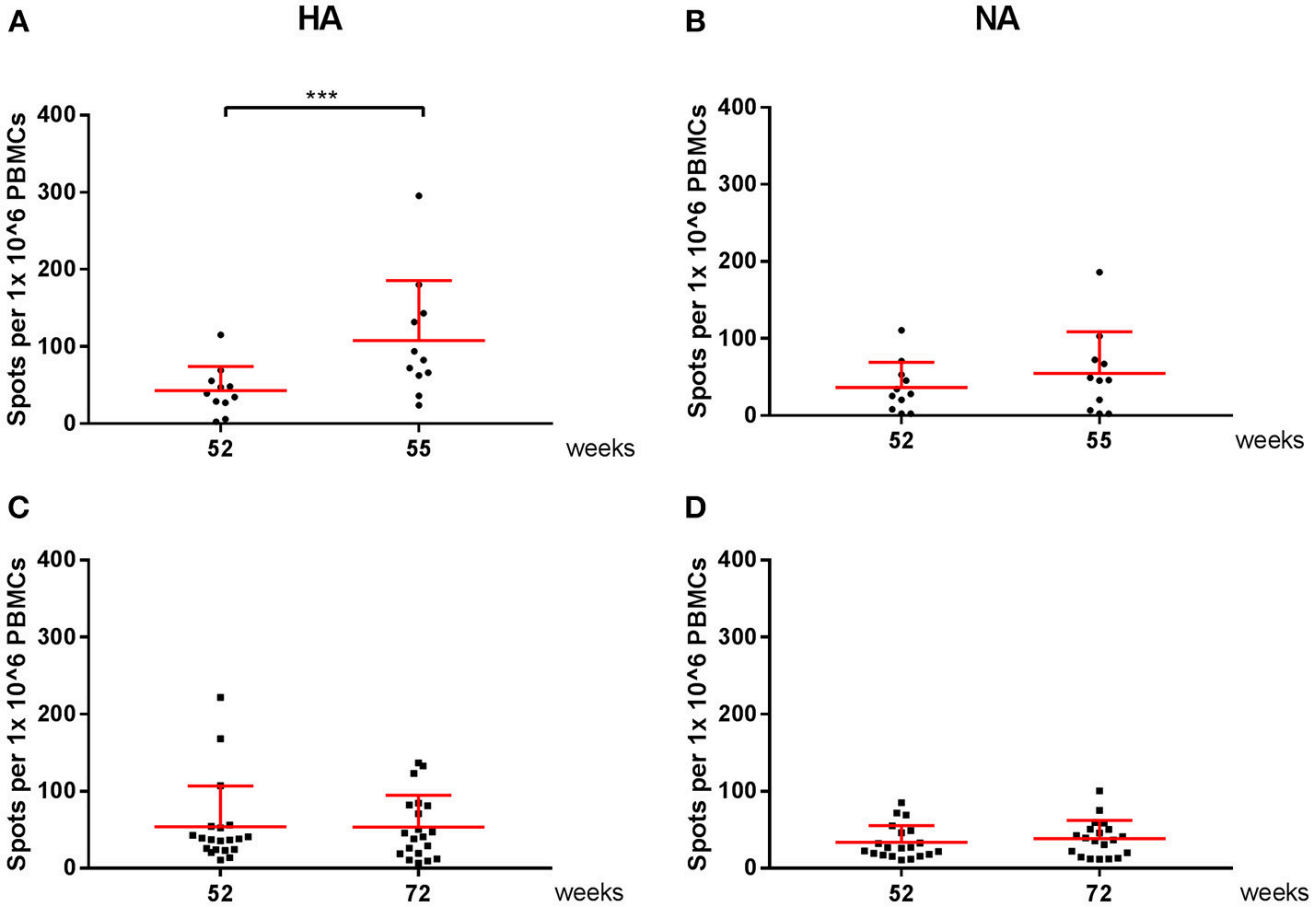
H3N2 virus-specific cellular responses in season 2. Responses against peptide pools of A/Perth/16/2009(H3N2) HA **(A,C)** and NA **(B,D)** were measured with an IFN-γ ELISpot in individuals of the vaccine **(A,B)** and control group **(C,D)**. In red the mean and SD of each data set is depicted. ELISpot data were analyzed with Wilcoxon matched-pairs rank test. ^***^P < 0.001.

Vaccine-specific cellular responses were also observed during season 2. Three weeks post seasonal vaccination, PBMCs of individuals in the vaccine and control groups were isolated and stimulated with HA or NA of both A(H1N1)pdm09 and H3N2 (Figures 6, 7). Vaccinated individuals showed increased responses to most peptide pools (*P* = 0.02; *P* < 0.0001 respectively; Figures 6A,B and *P* < 0.001 and *P* = NS, respectively; Figures 7A,B). Individuals in the control group had no significant induction of responses after stimulation with HA or NA derived from A(H1N1)pdm09 and HA of H3N2 (Figures 6C,D, 7C,D). Thus, we show that both the adjuvanted and the unadjuvanted vaccine are indeed capable of inducing HA and NA-specific cellular responses.

## Discussion

In a cohort of health professionals, we showed the induction of both humoral and cellular immunity by both MF59-adjuvanted and unadjuvanted subunit influenza vaccines. We performed mixed effects regression analysis to correct the data for possible confounders, such as pre-existing immunity due to influenza virus infection and vaccination, and age, and sex. This enabled the comparison of the antibody and T cell responses and led us to conclude that although there is a role for both the humoral and cellular arms of the immune system following influenza vaccination, they show a different profile of induction and waning.

In the Netherlands, the MF59-adjuvanted vaccine was used for risk groups in a two-dose schedule (10). The two-dose schedule was based on experience with H5 influenza vaccines for which two adjuvanted vaccine doses were required to obtain seroprotective antibody levels (35–38). We showed that one dose of the adjuvanted pandemic vaccine already induced a seroprotective antibody response, which is defined as an HI titer of 40 or higher (28, 29). This was also demonstrated in other target groups of 2009 pandemic vaccination such as infants, elderly, and immunocompromised individuals (39–41). Although a second dose of the vaccine further increased antibody levels, the added value of this dose is unclear as 87.7% of the individuals had already reached a protective antibody level after one dose (data not shown). Therefore, we postulate that a second dose to boost the humoral response is mostly of value for low antigenic influenza virus strains or limited to no cross-protective immunity.

Primary analysis of vaccine-induced cellular responses was initially performed by stimulation of PBMCs with an influenza virus strain homologous to the strain used in the vaccine. Responses measured using this assay could be attributed to both the surface proteins of the virus but also to the internal proteins of the virus. To confirm that we are dealing with vaccine-specific T cell responses directed against the HA or NA surface proteins of influenza virus, we performed antigen-specific T cell assays using pools of 15-mer peptides, capable of stimulating both CD4^+^ and CD8^+^ T cells, covering the entire HA and NA surface proteins of the vaccine strain. We showed that the first dose of the pandemic vaccine induced a cellular response as described for MF59 and AS03-adjuvanted vaccines (22, 42). Interestingly, the second dose did not enhance the cellular immune response. A factor that could be considered for this lack of booster effect is the timing of the second vaccination and therefore the activation state of the T cells, since these cells are likely still activated, and thus may not be activated further.

Our model shows that an unadjuvanted seasonal subunit vaccine is capable of inducing a cellular response similar to that induced by the adjuvanted pandemic subunit vaccine. Here it has to be noted that the adjuvanted pandemic vaccine was monovalent, contained 7.5 μg HA and responses were measured 2 weeks after vaccination, while the seasonal vaccine was a trivalent vaccine containing 15 μg of each HA and responses were measured 3 weeks after vaccination. No significant difference was found between antibody levels of individuals that had received the first dose of the adjuvanted pandemic vaccine detected at week 3 (V_1_V_2_ and V_1_C_2_) and individuals vaccinated only in season 2 with a single dose of the seasonal vaccine detected at week 55 (C_1_V_2_) (Figure 5 and Supplemental Table 1D). However, it might be that the antigen dose compensated for the lack of adjuvant in the seasonal vaccine; which should be investigated further.

During the subsequent post-pandemic influenza season (2010–2011), we continued monitoring both the humoral and cellular response and showed that both arms were activated by the seasonal vaccine. The induction of a cellular response by an unadjuvanted subunit vaccine is especially interesting as of now little data is available on the induction of T cells by vaccines containing only HA and NA as viral antigens. Induction of a cellular response has previously been shown for unadjuvanted split seasonal influenza vaccination, but these vaccines contain internal influenza proteins known to have highly conserved sequences to which memory might have been generated during previous infections (43, 44). In line with the results found in this study, van der Most et al. showed that both an AS03-adjuvanted and an unadjuvanted monovalent A(H1N1)pdm09 vaccine were capable of inducing vaccine-specific cellular responses directed to the HA antigen (42). They showed using flow cytometry that the cellular responses could mostly be contributed to CD4^+^ T cells and to a lesser extent to CD8^+^ T cells.

At the end of both seasons, which spanned more than 15 months, antibody levels remained detectable in the circulation, while cellular levels had reduced back to baseline levels by that time in individuals who were only vaccinated in season 1 with the adjuvanted pandemic vaccine. This does not necessarily indicate that vaccine-specific T cells are no longer present as memory T cells might reside in (lymphoid) tissues instead of in circulation which is not reflected by measuring T cell responses in the blood (45–48). The presence and reactivation of these T cells, even when present in a low number, may have an effect on subsequent exposures.

Bodewes et al. found that annual vaccination with a seasonal vaccine hampers the development of influenza-specific CD8^+^ T cells in children, indicating that vaccination history also affects the development of T cell responses (49). A similar conclusion was provided by van der Most et al. in healthy adults (42). McElhany et al. described a negative correlation between antibody levels and cytokine ratios in older adults and proposed that a second vaccination might skew T cell responses to the production of IL-10, which limits CTL induction but is advantageous for antibody responses (50). Thus, even though a second dose might be advantageous for inducing antibodies, the effect on the cellular arm of the immune response should not be underestimated and future studies should include analyses of the quality of the T cell response after vaccination with unadjuvanted vaccines.

During the 2009 pandemic, data became available that individuals who had cross-reactive T cells available were partially protected against infection with the pandemic virus (51). The importance of T cells is especially clear in situations where low cross-protective neutralizing antibodies are observed, and shows the added value of inducing T cell responses by vaccination (15, 16, 52–54).

Limitation of our study is that we did not power the original study to be performed during two consecutive seasons and only had a limited number of individuals enrolled in the C_1_V_2_ arm. Especially for the HA and NA-specific in depth cellular analysis our samples were limiting. In addition, we were not able to link our immunological data to influenza virus infections. We did monitor all individuals for influenza-like illness. However, both the first pandemic season and the second season were very mild in the Netherlands and only sporadic infections were observed in individuals in our study.

Concluding, the findings in this study have key implications for influenza vaccination strategies, especially when preparing for a pandemic. In most scenarios, one dose of the vaccine is sufficient to provide protection. Only if no cross-protective immunity is available or if the immunogenicity of the vaccine antigens is insufficient, two doses of a vaccine are warranted. As repeated influenza vaccination may not be favorable for the induction and quality of cellular responses, the number of doses to be administered should be carefully considered. To gain more insight into the mechanism of action behind these findings, studies describing the immune response following influenza vaccination should not only focus on the humoral immune response, but should also include analysis of cellular responses. Due to their considerable role in cross-protective immunity, there should also be more emphasis on inducing favorable cellular responses in influenza vaccine design.

## Ethics Statement

This study was carried out in accordance with the recommendations of Good Clinical Practice and the Declaration of Helsinki. All subjects gave written informed consent. The protocol was approved by the Central Committee on Research Involving Human Subjects of the Netherlands.

## Author Contributions

SR, NR, WL, and JvB conceptualized the study. RJ, JM-O, RvB, and JvB performed the clinical trial. SR, MH, RJ, and AW executed the laboratory experiments. SR, JvB, and JvdK performed the statistical analysis. SR, MH, WL, and JvB interpreted the data and wrote the manuscript. All authors critically revised the manuscript.

### Conflict of Interest Statement

The authors declare that the research was conducted in the absence of any commercial or financial relationships that could be construed as a potential conflict of interest.
