# Supplementary material for: Immunogenicity of Influenza Vaccines: Evidence for Differential Effect of Secondary Vaccination on Humoral and Cellular Immunity
Source: Front Immunol. 2019 Jan 29;9:3103. doi: 10.3389/fimmu.2018.03103 (PMC6362424; doi:10.3389/fimmu.2018.03103)
Supplement: Supplementary file 1 [file Data_Sheet_1.pdf]

Supplemental table 1A: Serological responses Season 1

**Vaccination response Season 1**		**RR *(2.5-97.5% CI)***	**p-value**
First dose *(week 3)*	Primary Baseline *(week 0)*	17.3 *(15.5-19.4)*	<0.001
Second dose *(week 6)*	Primary Baseline	21.7 *(19.3-24.2)*	<0.001
Contraction V *(week 26)*	Primary Baseline	11.1 *(9.8-12.4)*	<0.001
Second dose *(week 6)*	First dose *(week 3)*	1.3 *(1.1-1.4)*	0.001
Second dose *(week 6)*	Contraction V_1 *(week 26)*	0.5 *(0.5-0.6)*	<0.001
Contraction V_1 *(week 26)*	Maintenance V_1 *(week 52)*	1.5 *(1.3-1.7)*	<0.001

Supplemental table 1B: Serological responses Season 2

**Vaccination response Season 2**		**RR *(2.5-97.5% CI)***	**p-value**
Maintenance V_1 *(week 52)*	Primary Baseline	7.6 *(6.8-8.5)*	<0.001
Secondary seasonal Season 2 V_1V_2 *(week 55)*	Primary Baseline C_1C_2	24.4 *(21.6-27.5)*	<0.001
Contraction V_1V_2 *(week 72)*	Primary Baseline	24.4 *(21.6-27.4)*	<0.001
Residual V_1C_2 *(week 72)*	Primary Baseline	7.3 *(5.8-9.0)*	<0.001
Primary seasonal Season 2 C_1V_2 *(week 55)*	Primary Baseline	12.8 *(8.0-19.3)*	<0.001
Contraction C_1V_2 *(week 72)*	Primary Baseline	5.9 *(3.7-8.9)*	<0.001
Maintenance V_1 *(week 52)*	Secondary seasonal Season 2 V_1V_2 *(week 55)*	3.2 *(2.8-3.6)*	<0.001
Secondary seasonal Season 2 V_1V_2 *(week 55)*	Contraction V_1V_2 *(week 72)*	0.6 *(0.5-0.7)*	<0.001
Maintenance V_1 *(week 52)*	Residual V_1C_2 *(week 72)*	1.0 *(0.8-1.2)*	0.699
Primary seasonal Season 2 C_1V_2 *(week 55)*	Contraction C_1V_2 *(week 72)*	0.5 *(0.3-0.8)*	0.032

Supplemental table 1A: Serological responses Season 1

Vaccination response Season 1		RR *(2.5-97.5% CI)*	p-value
First dose *(week 3)*	Primary Baseline *(week 0)*	17.3 *(15.5-19.4)*	<0.001
Second dose *(week 6)*	Primary Baseline	21.7 *(19.3-24.2)*	<0.001
Contraction V *(week 26)*	Primary Baseline	11.1 *(9.8-12.4)*	<0.001
Second dose *(week 6)*	First dose *(week 3)*	1.3 *(1.1-1.4)*	0.001
Second dose *(week 6)*	Contraction V_1 *(week 26)*	0.5 *(0.5-0.6)*	<0.001
Contraction V_1 *(week 26)*	Maintenance V_1 *(week 52)*	1.5 *(1.3-1.7)*	<0.001

Supplemental table 1B: Serological responses Season 2

Vaccination response Season 2		RR *(2.5-97.5% CI)*	p-value
Maintenance V_1 *(week 52)*	Primary Baseline	7.6 *(6.8-8.5)*	<0.001
Secondary seasonal Season 2 V_1V_2 *(week 55)*	Primary Baseline C_1C_2	24.4 *(21.6-27.5)*	<0.001
Contraction V_1V_2 *(week 72)*	Primary Baseline	24.4 *(21.6-27.4)*	<0.001
Residual V_1C_2 *(week 72)*	Primary Baseline	7.3 *(5.8-9.0)*	<0.001
Primary seasonal Season 2 C_1V_2 *(week 55)*	Primary Baseline	12.8 *(8.0-19.3)*	<0.001
Contraction C_1V_2 *(week 72)*	Primary Baseline	5.9 *(3.7-8.9)*	<0.001
Maintenance V_1 *(week 52)*	Secondary seasonal Season 2 V_1V_2 *(week 55)*	3.2 *(2.8-3.6)*	<0.001
Secondary seasonal Season 2 V_1V_2 *(week 55)*	Contraction V_1V_2 *(week 72)*	0.6 *(0.5-0.7)*	<0.001
Maintenance V_1 *(week 52)*	Residual V_1C_2 *(week 72)*	1.0 *(0.8-1.2)*	0.699
Primary seasonal Season 2 C_1V_2 *(week 55)*	Contraction C_1V_2 *(week 72)*	0.5 *(0.3-0.8)*	0.032

Supplemental table 1C: Serological responses compared between groups

Comparison groups		RR *(2.5-97.5% CI)*	p-value
Secondary seasonal Season 2 V_1V_2 *(week 55)*	Primary seasonal Season 2 C_1V_2 *(week 55)*	2.0 *(1.2-3.1)*	0.028
Contraction V_1V_2 *(week 72)*	Contraction C_1V_2 *(week 72)*	0.5 *(0.4-0.6)*	<0.001
Contraction V_1V_2 *(week 72)*	Residual V_1C_2 *(week 72)*	2.0 *(1.7-2.6)*	<0.001
Residual V_1C_2 *(week 72)*	Contraction C_1V_2 *(week 72)*	1.3 *(0.8-2.1)*	0.699

Supplemental table 1D: Serological responses compared between time points

Comparison time points		RR *(2.5-97.5% CI)*	p-value
First dose *(week 3)*	Primary seasonal 2010-2011 C_1V_2 *(week 55)*	0.7 *(0.5-1.1)*	0.599
Second dose *(week 6)*	Secondary seasonal Season 2 V_1V_2 *(week 55)*	1.1 *(1.0-1.3)*	0.328
Maintenance V_1 *(week 52)*	Contraction C_1V_2 *(week 72)*	0.7 *(0.5-1.2)*	0.674

Supplemental table 1E: Influenza H3N2 virus-specific serological responses in season 2

		RR *(2.5-97.5% CI)*	p-value
Vaccination	Baseline	4.7 *(3.9-5.6)*	<0.001
Vaccination	Contraction	1.8 *(1.5-2.20)*	<0.001
Contraction	Baseline	2.6 *(2.2-3.1)*	<0.001

Supplemental table 2A: Influenza A(H1N1)pdm09 virus-specific cellular responses season 1

**Vaccination response Season 1**		**RR** ***(2.5-97.5% CI)***	**p-value**
First dose *(week 2)*	Primary Baseline	1.5 *(1.3-1.7)*	<0.001
Second dose *(week 6)*	Primary Baseline	1.4 *(1.2-1.6)*	<0.001
Maintenance V_1 *(week 52)*	Primary Baseline	1.7 *(1.4-1.4)*	<0.001
First dose *(week 2)*	Second dose *(week 6)*	0.9 *(0.8-1.1)*	0.819
Second dose *(week 6)*	Maintenance V_1 *(week 52)*	1.2 *(1.0-1.4)*	0.11

Supplemental table 2B: Influenza A(H1N1)pdm09 virus-specific cellular responses season 2

**Vaccination response Season 2**		**RR** ***(2.5-97.5% CI)***	**p-value**
Secondary seasonal Season 2 V_1V_2 *(week 55)*	Primary Baseline	2.5 *(2.0-3.0)*	<0.001
Primary seasonal Season 2 C_1V_2 *(week 55)*	Primary Baseline	2.2 *(1.5-3.1)*	<0.001
Residual V_1C_2 *(week 72)*	Primary Baseline	1.2 *(0.9-1.4)*	0.544
Maintenance V_1 *(week 52)*	Secondary seasonal Season 2 V_1V_2 *(week 55)*	1.5 *(1.2-1.8)*	0.001
Maintenance V_1 *(week 52)*	Residual V_1C_2 *(week 72)*	0.7 *(0.6-0.9)*	0.005

Supplemental table 2C: Comparison groups influenza A(H1N1)pdm09 virus-specific cellular responses

**Comparison groups**		**RR** ***(2.5-97.5% CI)***	**p-value**
Secondary seasonal Season 2 V_1V_2 *(week 55)*	Primary seasonal Season 2 C_1V_2 *(week 55)*	1.2 *(0.8-1.7)*	0.819
Secondary seasonal Season 2 V_1V_2 *(week 55)*	Residual V_1C_2 *(week 72)*	2.1 *(1.7-2.7)*	<0.001
Primary seasonal Season 2 C_1V_2 *(week 55)*	Residual V_1C_2 *(week 72)*	1.9 *(1.2-2.8)*	0.021

Supplemental table 2D: Influenza H3N2 virus-specific cellular responses in season 2

		**RR** ***(2.5-97.5% CI)***	**p-value**
Vaccination	Baseline	1.9 (1.6-2.2)	<0.001
