# Supplementary material for: Immunogenicity of Influenza Vaccines: Evidence for Differential Effect of Secondary Vaccination on Humoral and Cellular Immunity
Source: Front Immunol. 2019 Jan 29;9:3103. doi: 10.3389/fimmu.2018.03103 (PMC6362424; doi:10.3389/fimmu.2018.03103)
Supplement: Supplementary file 2 [file Data_Sheet_2.pdf]

Legend Supplemental Figure 1: HI titers of H3N2 virus specific antibodies and IFN-γ-specific responses of H3N2 virus-stimulated and SEB-stimulated PBMCs by ELISpot.

Geometric mean titer (GMT) with SD of A(H3N2)-specific antibodies in vaccinated individuals and individuals of the control group of the per protocol group during season 2 (A). Antibody responses were tested with paired T test for longitudinal samples of individuals in the same group and unpaired T test for analysis of samples from different groups.
■ vaccinated □ controls … protective antibody level of 40 ****p<0.0001 N.A. not applicable
Spots per million PBMCs of A(H3N2)-specific (B) and SEB-induced (C) IFN-γ responses by ELISpot in vaccinated individuals and individuals of the control group during season 2. In red the mean and SD of each data set is depicted.
ELISpot data were analyzed with Wilcoxon matched-pairs rank test. ●vaccinated, ■ controls
* p<0.05, ** p<0.01, ***p<0.001
