# Supplementary figures and images for: Immunogenicity of Influenza Vaccines: Evidence for Differential Effect of Secondary Vaccination on Humoral and Cellular Immunity
Source: Front Immunol. 2019 Jan 29;9:3103. doi: 10.3389/fimmu.2018.03103 (PMC6362424; doi:10.3389/fimmu.2018.03103)

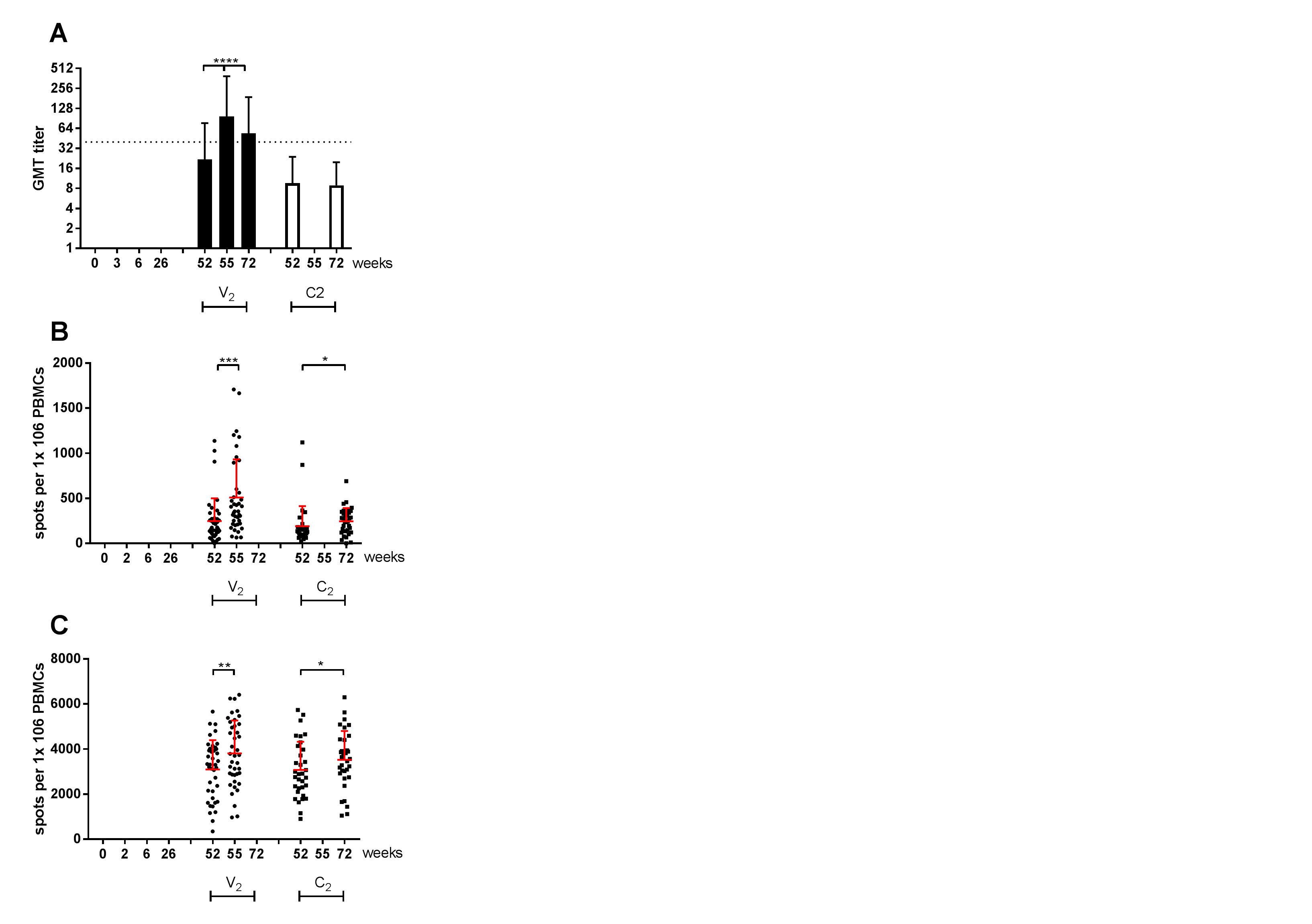

Supplement: Supplementary file 3 [file Image_1.TIF]
